# Bionomic response of *Aedes aegypti* to two future climate change scenarios in far north Queensland, Australia: implications for dengue outbreaks

**DOI:** 10.1186/1756-3305-7-447

**Published:** 2014-09-19

**Authors:** Craig R Williams, Gina Mincham, Scott A Ritchie, Elvina Viennet, David Harley

**Affiliations:** Sansom Institute for Health Research, University of South Australia, GPO Box 2471, Adelaide, 5001 Australia; School of Public Health, Tropical Medicine and Rehabilitation Sciences, James Cook University, Cairns, Queensland Australia; National Centre for Epidemiology and Population Australian National University, Canberra, Australia

## Abstract

**Background:**

Dengue viruses are transmitted by anthropophilic mosquitoes and infect approximately 50 million humans annually. To investigate impacts of future climate change on dengue virus transmission, we investigated bionomics of the mosquito vector, *Aedes aegypti*.

**Methods:**

Using a dynamic life table simulation model (the Container inhabiting mosquito simulation CIMSiM) and statistically downscaled daily values for future climate, we assessed climate change induced changes to mosquito bionomics. Simulations of *Ae. aegypti* populations for current (1991-2011) and future climate (2046-2065) were conducted for the city of Cairns, Queensland, the population centre with most dengue virus transmission in Australia. Female mosquito abundance, wet weight, and the extrinsic incubation period for dengue virus in these mosquitoes were estimated for current and future climate (MPI ECHAM 5 model, B1 and A2 emission scenarios).

**Results:**

Overall mosquito abundance is predicted to change, but results were equivocal for different climate change scenarios. *Aedes aegypti* abundance is predicted to increase under the B1, but decrease under the A2 scenario. Mosquitoes are predicted to have a smaller body mass in a future climate. Shorter extrinsic incubation periods are projected.

**Conclusions:**

It is therefore unclear whether dengue risk would increase or decrease in tropical Australia with climate change. Our findings challenge the prevailing view that a future, warmer climate will lead to larger mosquito populations and a definite increase in dengue transmission. Whilst general predictions can be made about future mosquito borne disease incidence, cautious interpretation is necessary due to interaction between local environment, human behaviour and built environment, dengue virus, and vectors.

## Background

Two point five billion people globally are at risk of dengue virus (DENV) infection, transmitted by *Aedes* mosquitoes mostly in urban and peri-urban areas. Approximately 50 million DENV infections occur annually, with a disease burden of around 1300 disability-adjusted life years [1].

The principal DENV vectors are *Aedes aegypti* (L.) and *Aedes albopictus* (Skuse); mosquitoes that utilize water-filled structures in human settlements for breeding. The contribution of rainfall, humidity and temperature to the provision of mosquito habitat and the growth and development of these ectotherms means there is a strong link between climate and dengue disease, and changes to climate are projected to alter future transmission. A rise in global mean surface temperature of 0.4-2.6°C has been forecast for the mid-21^st^ century, with spatially heterogeneous changes to precipitation and sea level [2]. Thus, the intensity and distribution of mosquito-borne diseases, including dengue, is expected to alter in a future climate.

A number of studies predicting changes to future disease range and intensity exist, and there is a body of evidence that suggests increased geographic range for dengue virus transmission in a future climate. Some of these are based on the relationships between climate variables and disease incidence. There are models that describe a positive relationship between notified dengue cases and increasing temperature [3] and humidity [4]. Based on these models projected climate change has been predicted to increase dengue transmission by expansion of geographic range [3–5] and increased transmission intensity and seasonal duration in areas already at risk [3, 6]. Generally a global increase in the ability of mosquitoes to transmit dengue viruses has been predicted. But more recently the impact of social factors such as vector control, housing quality and income has been predicted to mitigate dengue risk. Use of a statistical model incorporating gross domestic product demonstrated that climate change effects alone would increase the probability of dengue transmission, but increasing gross domestic product will decrease future risk [5].

The geographic range of dengue has generally decreased in recent decades [7], particularly in developed nations such as Australia [8, 9], despite a warming climate since the mid 20^th^ century [2]. Recent warming climate has not been associated strongly with increased dengue transmission [6].

Superficially observations and projections are discordant. This may be attributable in part to the simplicity of dengue models. Statistical models that relate climate variables to disease presence and incidence over large geographic areas may produce projections that are in error, as they are based on historic associations between weather and disease. Conversely, models based on observations from smaller spatial scales have the facility to incorporate locally-relevant aspects of dengue ecology, as do models that incorporate mechanistic processes determining the ecology of the vector [10]. Mechanistic modeling with the dengue simulation model (DENSiM) has been used to model inter-annual variability in dengue activity in Queensland [11], demonstrating that local meteorological phenomena impact dengue transmission.

Relationships between temperature and mosquito fitness have been established for a number of mosquito species [12], and thermal optima (ranges of temperatures at which fitness is maximized) have been established. In general, climate warming is expected to favour mosquitoes that have broad thermal optima and can adapt to higher temperatures [12]. Studies of reproduction of *Ae. aegypti* indicate that it will adapt well to moderate increases in temperature [13].

The relationship between climate and DENV vector mosquito productivity and bionomics has not been investigated. There have been predictions that climate change will increase the abundance of disease vectors because mosquitoes will develop faster and complete more generations annually, and that warmer temperatures will decrease virus incubation time [12], with both phenomena contributing to increased dengue incidence. However, these predictions have not been supported by quantitative evidence from mosquito population dynamics models.

As part of a larger study to understand the impact of future climate change on dengue transmission in Australia, we investigated likely changes to *Ae. aegypti* bionomics under current and future climate. Climate has changed through time and will continue to do so. Further, changes to society, economy, housing, and travel patterns will also go on. Therefore, the causes for change in dengue epidemiology are many, and disentangling the predominant risk determinants is challenging.

In an effort to do this, we investigated how mosquito abundance, body size and virus incubation rate are projected to alter utilizing a field-validated mechanistic mosquito population model and climate projections. This was done for an area of annual dengue transmission and high receptivity to dengue importation, Cairns, in Far North Queensland, Australia. This location is the hub of local dengue transmission in Australia, with the largest epidemic occurring there in 2008-2009 (over 900 cases and one death) [14]. Given the history of dengue vector and epidemiology studies performed there, Cairns presents an ideal location to investigate potential impacts of climate change on dengue vector bionomics.

## Methods

### The Container-inhabiting Mosquito Simulation (CIMSiM)

The modelling software CIMSiM 3.27 (University of California) was used to simulate *Ae. aegypti* population dynamics. CIMSiM [15] accurately models *Ae. aegypti* population dynamics in Queensland [16], and has been used to study *Ae. aegypti* productivity [9, 17] and persistence [18]. CIMSiM was also used to provide entomological data for modeling inter-annual variation in dengue transmission in Australia [11] using the DENSiM (dengue simulation) model attached to it [19]. CIMSiM generates daily estimates of egg, larval, pupal and adult numbers per hectare by integrating daily meteorological observations with information about available breeding habitats. In this way, CIMSiM can be applied to any locality in which the container type and frequency are known and for which meteorological data are available.

The container types used in the model represented six different breeding habitats available to *Ae. aegypti*: tyres, buckets, tarpaulins, pot plant bases, rainwater tanks and drain sumps (Table 1). In the simulation, the dimensions of containers described in Table 1 are used to calculate water depth each day, and describe the filling and emptying of containers in response to rainfall and evaporation. Water flux for these containers has been field calibrated by matching water depth in the field with simulations, and adjusting the ‘sun exposure’ and ‘water shed’ values (as in Table 1) accordingly [16]. Whilst in reality containers are a variety of sizes, the dimensions selected here represent typical dimensions encountered in the field. The food delivered to larvae (Table 1) was also calibrated against field data [16] or previously published data [20, 21] by adjusting food values in the model until pupal productivity matched that of field observations. Container densities per hectare (Table 1) were typical values derived from unpublished field survey data (PH Johnson, pers. comm. 2009, James Cook University) and previous publications [16, 17].

**Table 1:** **Breeding container parameters as used in** ***Aedes aegypti*** **population modelling using CIMSiM**

	Buckets	Pot plant saucers	Tarpaulins	Tyres	Subterranean	Rainwater tanks
Dimensions (cm)	18.0 d, 17.4 h	6.1 × 2.0 × 26.0	15.5 × 22.6 × 4.2	35.3 d, 11.0 w	64.6 × 72.7 × 172.0	200 d × 200 h
Capacity	4.87 L	0.61 L	0.52 L	5.3 L	807.8 L	6,284.0 L
Sun exposure	0.2	0	0.8	0.3	0.9	0.5
Container cover	0	1	0	1	1	1
Water shed ratio	1.2	0	0.6	0.2	10	10
Draw down (L)	0	0.3	0	0	0	628.4 L
Initial food (mg)	70	100	450	500	144.75	50
Food/d (mg)	30	10	175	100	144.75	50
density per ha.	1.0	1.0	0.5	0.5	0.1	0.1

### Meteorological data for current and future climates

Carbon cycle models generally give estimates of atmospheric carbon dioxide concentrations ranging from 500 to 1200 ppm for the mid 21^st^ century compared to concentrations of 352 ppm in 1990 [2]. The two emission scenarios A2 and B1 differ in their estimated atmospheric carbon dioxide concentration and consequent temperature rises. The A2 emission scenario is estimated to lead to an atmospheric carbon dioxide concentration between 372 and 572 ppm. This scenario assumes a relatively slower transition to renewable energy sources with consequent high carbon emissions, and likely global temperature increases from 2.0-5.4°C by the end of the 21^st^ C [2]. In comparison the B1 emission scenario assumes carbon dioxide concentrations will stabilise at 550 ppm by the mid-21^st^ century. The B1 climate scenario includes rapid economic growth with a population rise to 9 billion in 2050, with slow progression towards a service and information economy with some reductions in material intensity, with likely global temperature increases from 1.1-2.9°C by the end of the 21^st^ C [2]. These scenarios were chosen to reflect a range of possible futures with varying carbon emissions and temperature rises to determine the likely impact of future mitigation strategies on mosquito bionomics.

Projections for the A2 and B1 scenarios for Cairns Queensland were statistically downscaled to daily data (by the Bureau of Meteorology, Australia) and imported into CIMSiM. To gain an indication of overall mean changes in weather, daily averages for each variable were calculated, as was the projected change from current climate (Table 2).

**Table 2:** **Daily average meteorological values (standard deviation in parentheses) for Cairns, Queensland for both current climate and future climate (MPI ECHAM 5 model) under two emissions scenarios**

Climate Scenario	Max T (°C)	Ave T (°C)	Min T (°C)	Rain (mm)	RH (%)
Current (1990-2011)	29.27 (2.54)	25.11 (2.64)	20.95 (3.23)	5.50 (18.91)	73.76 (8.92)
Future B1 (2046-65)	29.51 (2.76)	25.71 (2.47)	21.90 (2.63)	6.91 (19.43)	76.62 (7.34)
change	+0.24°C	+0.6°C	+0.95°C	+1.41 mm	+2.86%
Future A2 (2046-65)	29.45 (2.80)	25.70 (2.47)	21.95 (2.57)	6.22 (20.31)	76.6 (7.41)
change	+ 0.18°C	+0.59°C	+1.00°C	+0.72 mm	+2.84%

### Simulations

For a focal site of dengue transmission in tropical Australia (Cairns, Queensland), female mosquito abundance, wet weight, and the extrinsic incubation period for dengue virus in *Ae. aegypti* were estimated using simulations for the recent climate (1990-2011, as an estimate for ‘current climate’) and for both future climate change scenarios using the MPI ECHAM 5 model for the period 2046-2064). The monthly averages for each biological parameter (adult female mosquito abundance, wet weight) were calculated based on CIMSiM outputs. Extrinsic Incubation Period (EIP) of dengue virus in the mosquitoes was obtained for simulation periods using the DENSiM module attached to CIMSiM 3.27. Thirty replicate simulation runs were performed for each scenario.

### Statistical analysis

Monthly data for abundance, wet weight and EIP were compared between the three scenarios (current climate and two future climate scenarios) using Analysis of Variance (STATA Ver 11. Statacorp, College Station TX). Bartlett’s test was used to check for homogeneity of variance [22] and Scheffe’s test was used *a posteriori* to determine which scenarios differed [23]. Comparisons were made for the months of January (wet season, during which most DENV transmission occurs in Cairns) and July (dry season, typical low point in transmission).

## Results

Average temperature increase of about 0.6°C and approximately 1 mm additional rain per day are projected for Cairns 2046-2064. These projections are not significantly different between the A2 and B1 scenarios, although less rainfall increase was predicted for the A2 scenario (Table 2).

**Figure 1:**
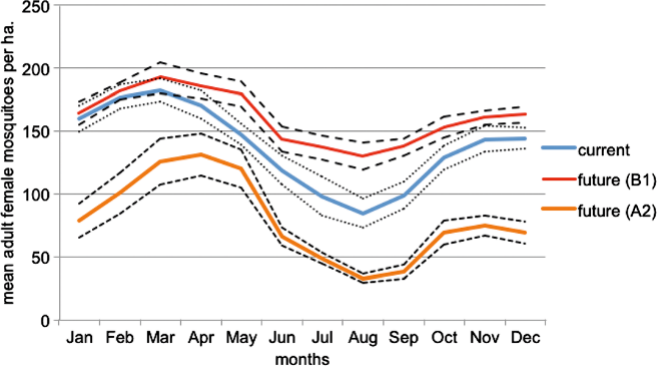
**Mean monthly female** ***Ae. aegypti*** **abundance for Cairns, Queensland from computer simulations for current (1990-2011) and future climate (2046-64) (dashed lines indicated 95% confidence intervals).**

Monthly mean *Ae. aegypti* female mosquito abundance (the number of mosquitoes present each day of a given month per hectare) was estimated for Cairns Queensland for both current climate, and two future climate scenarios (A2 and B1). The B1 scenario resulted in an increase in mosquito abundance throughout the year (16.6% on average monthly abundance), which was most obvious in the dry season (June – October) (Figure 1). Conversely, the A2 scenario resulted in a marked decrease in the average number of *Ae. aegypti* in all months (42.3% decrease). ANOVA revealed a significant difference in predicted abundance for January, with the A2 scenario predicting significantly fewer mosquitoes (F = 70.83, d.f. 54,2, *P* < 0.0001). The scenario-based vector population projections differed from one another in July (F = 54.14, d.f. 54,2, *P* < 0.0001).

**Figure 2:**
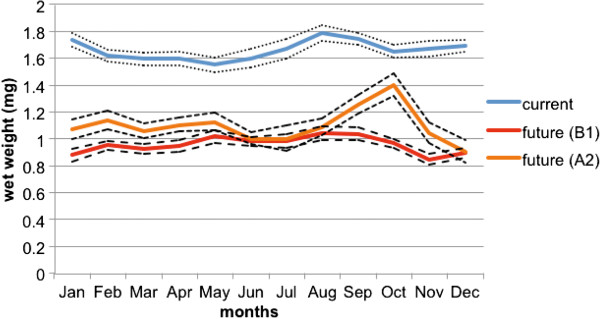
**Mean monthly female** ***Ae. aegypti*** **wet weight for Cairns, Queensland from computer simulations for current (1990-2011) and future climate (2046-64) (dashed lines indicated 95% confidence intervals).**

Body weight of mosquitoes was predicted to decrease in a future climate, for both the B1 and A2 scenarios (Figure 2). The B1 scenario was predicted to cause the largest decrease, from 1.67 mg in the current climate to 0.96 mg (a 42.5% decrease). Similarly, the A2 scenario was predicted to lead to average future wet weights of 1.1 mg (a 34.1% decrease). To relate these weights to wing length measurements (which are more commonly utilized as measures of mosquito size by researchers), we utilised the regression reported by Siegel *et al*. [24] from data of Christophers [25]. The predicted reductions in body mass equates to wing length 3.08 mm in the current climate, reducing to 2.6-2.7 mm in the future. Importantly, the current climate mosquito size calculated here (3.08 mm wing length) is very similar to that reported from previous field collections in the Cairns region (3.01 mm) [26].

ANOVA revealed a significant difference in wet weight for January, with differences between the current and both future scenarios evident (F = 224.66, d.f. 54,2, *P* < 0.0001). In July there was no difference in wet weights of the two future climate scenarios, with both being significantly less than that predicted for current climate (F = 99.00, d.f. 54,2, *P* < 0.0001).

**Figure 3:**
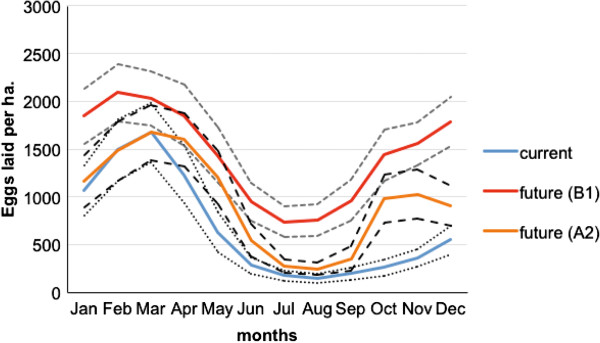
**Mean** ***Ae. aegypti*** **oviposition in all container types for Cairns, Queensland from computer simulations for current (1990-2011) and future climate (2046-64) (dashed lines indicated 95% confidence intervals).**

However, this reduction in body size was not linked to a reduction in oviposition rates, which were predicted to increase for both B1 and A2 scenarios (Figure 3).

**Figure 4:**
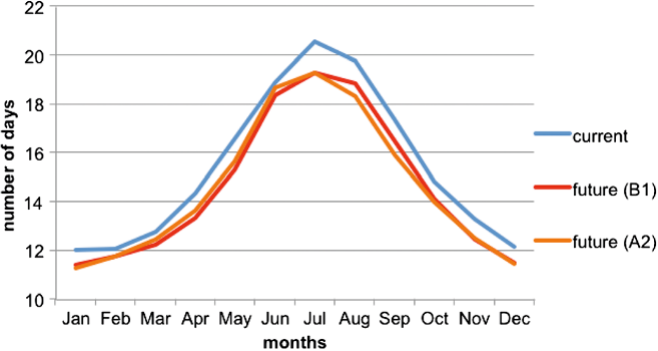
**Mean extrinsic incubation period (EIP) for DENV in** ***Ae. aegypti*** **for Cairns, Queensland from computer simulations for current (1990-2011) and future climate (2046-64).**

Dengue virus extrinsic incubation period was predicted to decrease in a future climate (Figure 4). Mean annual EIP was 15.4 d for the current climate, and was predicted to decrease by 5% for both scenarios (to 14.6d for B1, to 14.5 for A2). ANOVA revealed that both future climate scenarios had significantly shorter EIPs than for the current climate in January (F = 11.18, d.f. 54,2, *P* = 0.0001) and July (F = 11.18, d.f. 54,2, *P* = 0.0001).

## Discussion

Changes to future climate have been predicted to influence mosquito-borne disease incidence, primarily through alterations to mosquito ecology and pathogen incubation rates and survival. To characterize such changes for tropical Australia, we used a mechanistic modeling approach to explore changes to *Ae. aegypti* bionomics and dengue virus incubation rate.

A warmer and more humid climate will not necessarily increase mosquito abundance. Predictions for future mosquito abundance are inconsistent, with an increase or decrease under two scenarios (B1) and A2, respectively). Given the uncertainty in magnitude of temperature change under future climate, it makes sense to interpret these as equivocal results, neither predicting an increase or a decrease. A decrease can be predicted with climate warming indicating that simplistic extrapolation based on models incorporating few biological and environmental factors may lead to spurious conclusions regarding vector abundance, and therefore disease incidence.

Faster development rates enabling a greater number of generations to be completed per year may result from increased temperature. However, higher temperatures may lead to decreased abundance by increasing the probability of exceeding the optimal temperature range for growth and development, and thereby decreasing survival probabilities. Oviposition rates are predicted to increase under both future climate scenarios (Figure 3), and water levels for rain-filled containers are not predicted to decrease (data not shown). Therefore, under the range of climate encompassed by our modelling, mosquito abundance in a future climate is related to adult survivorship and the probability of successful completion of larval development.

The projected decrease in mosquito abundance under the A2 scenario may seem counter-intuitive, particularly given the projected increase in abundance for the B1 scenario (Figure 1). Higher temperatures are expected to increase the rate of larval development and boost mosquito populations, but if this is not matched by sufficient rainfall (less rainfall increase is predicted for the A2 scenario), the probability of eggs being hatched may decrease. For *Ae. aegypti,* eggs are laid at the waterline of containers, and high temperatures will cause greater evaporation of this water. Eggs are only hatched once the water level reaches that level again. An increase in temperature not matched by a sufficient rainfall increase may be just one mechanism leading to reduced mosquito abundance under the A2 scenario.

We do not claim here that our modelled representations of mosquito ecology will identically match every possible permutation in the field. Rather, our varying results predicting increases and decreases in mosquito abundance demonstrate that under future climate, both results are possible. This calls into question the overarching assumption that a warmer climate will lead to greater mosquito abundance.

Our modelling predicts *Ae. aegypti* of smaller body size, regardless of the particular emission scenario. This was not surprising, as mosquitoes will develop more quickly in higher temperatures, and thus have less opportunity to acquire nutrients. The decreased body size in future climate was not quite as evident for the A2 scenario in the late dry season, however (Figure 2), the result of cooler October temperatures predicted for this scenario compared with the B1, permitting longer larval periods. For *Ae. aegypti,* past research has indicated both decreased [27] and increased [28] host feeding with small body size. Whilst fecundity declines with decreasing body size in *Ae. aegypti* [29], host contact rates are thought to increase [28]. However, the role of host defensive behavior in these studies was not taken into account. Anopheline mosquitoes with smaller body size take blood more frequently [30], but the contribution to malaria transmission is thought to decrease with smaller body size [31]. Our current climate simulated body sizes were consistent with those measured in the field in the same region; 3.08 mm simulated compared with 3.01 mm recorded from the field [26], giving us confidence in our predictions. However, notwithstanding our predictions of smaller *Ae. aegypti* in a future climate, it is worth noting that mosquito body sizes will vary in the field (standard error of 0.01-0.02 mm [26]). Thus, whilst we predict overall smaller *Ae. aegypti* in a future climate, variation will still persist.

Future dengue transmission intensity and risk will be significantly, though certainly not solely, determined by vector ecology. Greater mosquito abundance may increase mosquito-host contact and increase dengue transmission. With decreased extrinsic incubation period, dengue transmission risk would be expected to increase. However, decreased mosquito body size is likely to decrease blood feeding frequency and may reduce dispersal and survival, with consequent lowered dengue incidence. Further, decreased body size could enhance dengue virus infectivity in mosquitoes and dissemination probability [32]. Conversely, whilst decreased mosquito body size in *Ae. aegypti* does not appear to impact oviposition success [33] it is likely to decrease fecundity (at least in some months of the year) and have some impact on blood feeding frequency. Smaller body size is also likely to negatively impact dispersal and survival [29].

The vectorial capacity (VC) equation first proposed by Macdonald [34] describes the overall ability of a vector species to transmit a pathogen at a particular time and place, with extrinsic virus incubation duration in the mosquito related strongly to increases in VC. Based on these relations a decrease in virus incubation time as predicted here (Figure 4) would increase VC. However, contradictory findings on future mosquito abundance, coupled with smaller body size could both reduce VC.

## Conclusions

It is unclear whether dengue incidence will increase in a future climate for our case study location in tropical north Queensland, Australia, if projections are based solely on vector factors. Indeed, projections of future vector numbers and abundance does not lead, inevitably, to projected increase in dengue incidence, and that therefore models require greater sophistication (and perhaps wholly new approaches to determining future risk for vector-borne disease risks).

Future work should focus on modeling future dengue transmission risk using a mechanistic approach which takes into account these predicted changes in mosquito body size and virus incubation rate. The Dengue Simulation model (DENSiM), which utilises outputs from the CIMSiM model used here, could be used for this purpose. DENSiM has been previously used to model inter-annual dengue activity in Australia [11].

By refining predictions of how dengue transmission may alter in a future climate we are better placed to determine the likely impacts of climate change on human health, so that resources to manage disease can be most effectively allocated. Such information may also be used to garner support for carbon emission reduction strategies. The information presented in this paper permits a more refined examination of how climate change may alter dengue in Australia, and may add rigour to the process of developing adaptive strategies and may inform government policy change.
